# Dopamine Transporter/α-Synuclein Complexes Are Altered in the Post Mortem Caudate Putamen of Parkinson’s Disease: An In Situ Proximity Ligation Assay Study

**DOI:** 10.3390/ijms19061611

**Published:** 2018-05-30

**Authors:** Francesca Longhena, Gaia Faustini, Cristina Missale, Marina Pizzi, Arianna Bellucci

**Affiliations:** 1 Department of Molecular and Translational Medicine, University of Brescia, 25123 Brescia, Italy; f.longhena@unibs.it (F.L.); g.faustini004@unibs.it (G.F.); mariacristina.missale@unibs.it (C.M.); marina.pizzi@unibs.it (M.P.); 2 Laboratory of Personalized and Preventive Medicine, University of Brescia, 25123 Brescia, Italy

**Keywords:** dopamine transporter, α-synuclein, Parkinson’s disease, proximity ligation assay

## Abstract

Parkinson’s disease (PD) is characterized by the degeneration of the dopaminergic nigrostriatal neurons and the presence of Lewy bodies (LB) and Lewy neurites (LN) mainly composed of α-synuclein. By using the in situ proximity ligation assay (PLA), which allows for the visualization of protein-protein interactions in tissues to detect dopamine transporter (DAT)/α-synuclein complexes, we previously described that these are markedly redistributed in the striatum of human α-synuclein transgenic mice at the phenotypic stage, showing dopamine (DA) release impairment without a DAT drop and motor symptoms. Here, we used the in situ PLA to investigate DAT/α-synuclein complexes in the caudate putamen of PD patients and age-matched controls. They were found to be redistributed and showed an increased size in PD patients, where we observed several neuropil-like and neuritic-like PLA-positive structures. In the PD brains, DAT immunolabeling showed a pattern similar to that of in situ PLA in areas with abundant α-synuclein neuropathology. This notwithstanding, the in situ PLA signal was only partially retracing DAT or α-synuclein immunolabeling, suggesting that a large amount of complexes may have been lost along with the degeneration process. These findings reveal a DAT/α-synuclein neuropathological signature in PD and hint that synaptic alterations involving striatal DAT may derive from α-synuclein aggregation.

## 1. Introduction

The main histopathological hallmarks of Parkinson’s disease (PD) are the progressive loss of dopaminergic neurons of the substantia nigra *pars compacta*, which project into the striatum, and the presence of Lewy bodies (LB) and Lewy neurites (LN), protein inclusions mainly composed of α-synuclein [1,2]. Alpha-synuclein is a small presynaptic protein that is able to interact with other proteins at the dopaminergic synapse and can regulate the release of dopamine (DA) [3,4,5,6]. Previous studies reported the DA transporter (DAT) as one of the α-synuclein interactants [7,8,9,10,11,12,13]. In particular, a direct binding of α-synuclein to the C-terminal tail of DAT has been reported [12]. At the synaptic level, DAT controls the spatial and temporal dynamics of DA neurotransmission by driving reuptake of the extracellular neurotransmitter into presynaptic neurons [8,14,15,16]. The activity of the DAT is finely tuned by three mechanisms: post-translational modifications, intracellular trafficking, and protein-protein interactions [17]. The different localization of DAT on the plasma membrane or in the intracellular space is often modulated by interactions with trafficking and regulatory proteins such as α-synuclein [17]. Several studies have shown that α-synuclein can modulate the activity of DAT by regulating the presence of the transporter on the cell surface [7,16,18,19]. Although the exact activity of α-synuclein on DAT function is still matter of debate [20,21], most of the studies conducted on both in vivo and in vitro models support that α-synuclein acts as a negative regulator of DAT activity [8,15,16,18,19,22,23]. Interestingly, exogenous α-synuclein is able to reduce DA reuptake by acting on DAT, suggesting that the transporter could mediate the toxicity of extracellular α-synuclein [24]. In line with the marked nigrostriatal dopaminergic degeneration occurring in PD [25,26], we found that both α-synuclein and DAT levels are decreased in the caudate putamen of patients [27]. However, nigral α-synuclein burden has been found to correlate with striatal DAT loss, supporting that DAT levels are modulated by α-synuclein aggregation processes [28]. Moreover, previous findings by our group indicated the occurrence of marked alterations in the distribution of DAT/α-synuclein complexes occurring in parallel to early striatal synaptic deficits in an α-synuclein transgenic mouse model of PD [7,29]. Interestingly, this evidence is in line with other observations from our group supporting that DAT/α-synuclein co-redistribution within intracellular inclusions is an early consequence of α-synuclein aggregation and can be reverted by agents modulating DAT function and distribution such as DA D2/D3 receptor agonists and cocaine [10]. Here, we aimed to investigate the occurrence of DAT/α-synuclein complexes and their distribution in the caudate putamen of PD patients and age-matched controls. The formation of DAT/α-synuclein complexes was studied by using classical immunohistochemical methods and the in situ proximity ligation assay (PLA), a well-established technique that allows for the visualization of protein-protein interactions in the tissue [7,27,30,31,32,33].

## 2. Results

### 2.1. DAT Redistribution in the Caudate Putamen of Patients Affected by PD

**Figure 1:**
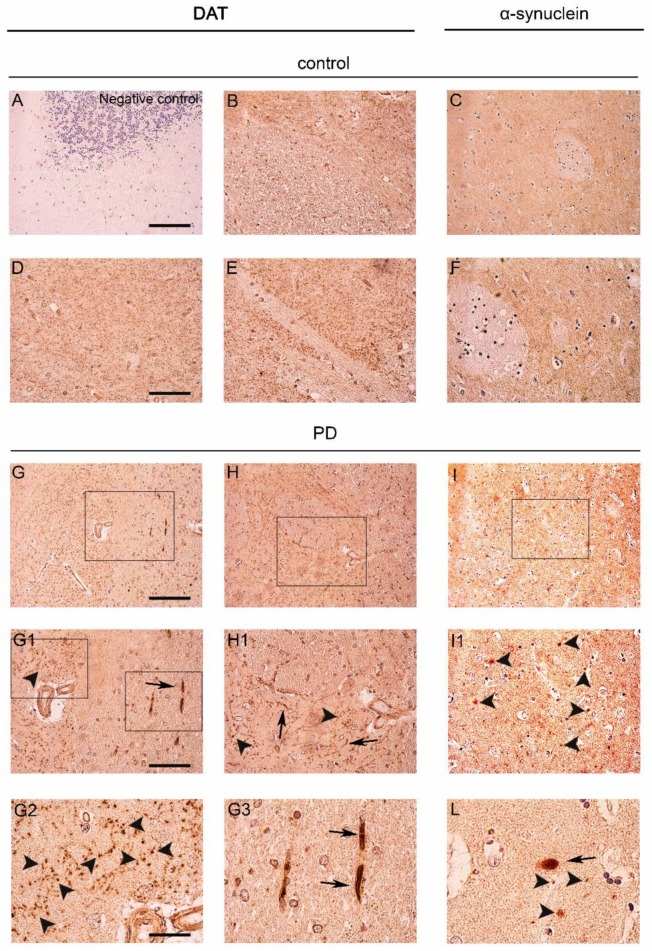
Dopamine transporter (DAT) staining in the caudate putamen of PD patients and age-matched controls. (**A**) Representative photomicrograph showing the negative control produced by performing DAT immunolabeling on cerebellum sections. Please note the absence of staining that is indicative of the specificity of the DAT antibody used for this study; (**B**,**D**,**E**,**G**,**H**,**G1**,**H1**,**G2**,**G3**) Representative images showing DAT immunolabeling in the caudate putamen of PD patients and age-matched controls subjects. DAT immunolabeling showed a small dot-like appearance with widespread distribution in the control sections (**B**,**D**,**E**). Please note that in spite of the overall decrease of immunolabeling, in the PD brains, the DAT signal was accumulated in big neuropil-like dots (**G**,**H**,**G1**,**H1**,**G2**, arrowheads) and in few neuritic-like structures (**G**,**G1**,**G3**, arrows); (**C**,**F**,**I**,**I1**,**L**) Panels are showing α-synuclein immunolabeling in the caudate putamen of PD patients and age-matched control subjects. Alpha-synuclein immunolabeling showed a widespread distribution in the grey matter in control sections (**C**,**F**). Please note the presence of neuropil-like α-synuclein-positive dots (**I**,**I1**,**L**, arrowheads) and LB-like structures in the PD brains (**L**, arrow). Scale bars: (**A**–**C**,**G**–**I**) 100 µm, (**D**–**F**,**G1**,**H1**,**I1**) 50 µm, (**G2**,**G3**,**L**) 20 µm.

The distribution of DAT signal was investigated in the caudate putamen of patients affected by PD and age-matched-controls. In the control subjects, the protein showed a diffuse staining in the white matter, with a very faint dot-like distribution (Figure 1B,D,E). On the other hand, in the caudate putamen of PD patients, we observed an apparent reduction of the diffuse punctate pattern of DAT (Figure 1G,H,G1,H1,G2,G3) when compared to the controls (Figure 1B,D,E). This decrease in the DAT signal in PD patients confirms our recent Western blot results [27] as well as previous DAT immunolabeling data [25,26,34,35].

This notwithstanding, DAT immunostaining was concentrated in thicker neuropil-like dots (Figure 1G,H,H1,H1,G2, arrowheads) and in swollen and filiform shape neurite-like structures (Figure 1G3, arrows). Specificity of the signal was confirmed by the absence of DAT immunolabeling in sections of the cerebellum of one of the control subjects (Figure 1A). Interestingly, the distribution of DAT staining appeared to retrace that observed for α-synuclein in PD patients and control subjects [27] where α-synuclein showed a sparse distribution in the caudate putamen of the controls (Figure 1C,F) and a widespread reduction with the presence of α-synuclein-positive LB (Figure 1L, arrow) and neuropil-like dots (Figure 1I,I1,L, arrowheads) in the PD subjects.

### 2.2. Co-Localization and Redistribution of DAT and α-Synuclein in the Caudate Putamen of PD Patients and Age Matched Controls

**Figure 2:**
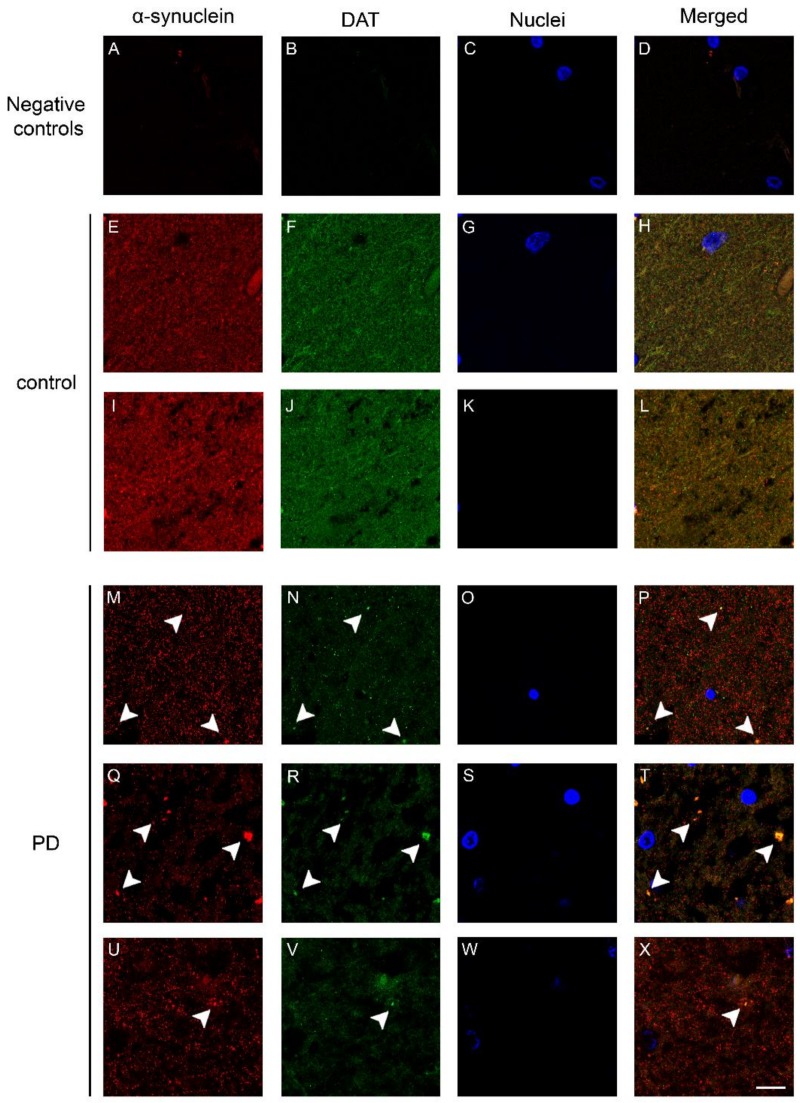
Double immunolabeling showing the DAT and α-synuclein in the caudate putamen of PD patients and control subjects. (**A**–**D**) Representative images showing negative controls performed by incubating slides with the secondary antibodies used for labeling either the DAT or α-synuclein only; (**E**–**X**) Panels show the confocal images of the DAT and α-synuclein fluorescent immunolabeling in the caudate putamen of patients affected by PD (**M**–**X**) and age-matched controls (control) (**E**–**L**). Please note the decrease of the widespread dot-like DAT immunolabeling in the PD samples that showed an accumulation of the protein in co-localization with α-synuclein in big clumps (**M**,**N**,**P**,**Q**,**R**,**T**,**U**,**V**,**X**, arrowheads). Scale bar: 20 µm.

**Figure 3:**
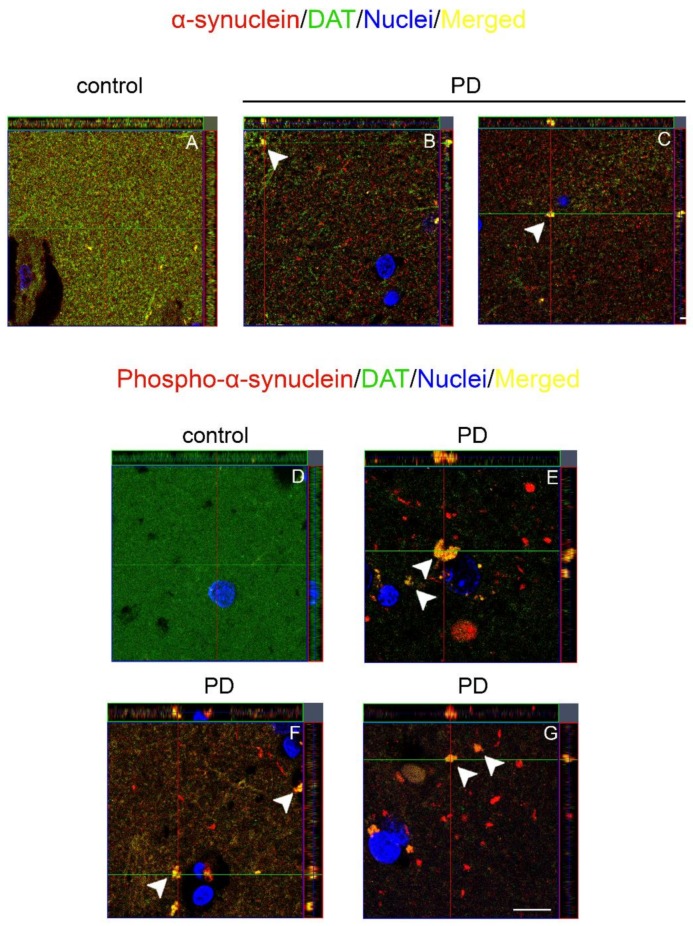
Orthogonal z reconstruction of α-synuclein/DAT and phosphor- α-synuclein/DAT immunolabeling. (**A**–**C**) Representative images of orthogonal z reconstruction of α-synuclein (red) and DAT (green) immunolabeling performed in the control subjects (**A**) and PD patients (**B**,**C**). Please note the presence of clumps that showed immunopositivity for both signals (**B**,**C**, arrowheads) in the caudate putamen of the brain of patients affected by PD; (**D**–**G**) Panels are showing the orthogonal z reconstruction of phospho-α-synuclein (red) and DAT (green) staining. Please note the absence of phosphorylated form of α-synuclein in the brain of control subjects (**D**), whereas in the brain of patients affected by PD, we observed the presence of phospho-α-synuclein/DAT positive clumps (**E**–**G**, arrowheads). Scale bars: (**A**–**C**,**D**–**G**) 20 µm.

Since we found some similarities in DAT and α-synuclein immunollabeling, we performed double fluorescence immunolabeling of the DAT and α-synuclein in order to investigate the presence of a co-localization of the proteins in the human brain. In the caudate putamen of the control subjects, DAT and α-synuclein immunolabeling exhibited a partial co-localization (Figure 2H,L), with a diffuse dot-like fashion reminiscent of the DAT and α-synuclein brown staining (Figure 1) [27], in line with our previous studies [36]. Of note, in the caudate putamen of patients affected by PD, we observed an apparent reduction of the DAT signal (Figure 2N,R,V) when compared with the control subjects (Figure 2F,J) in line with our previous Western blot results [27]. This notwithstanding, again we found the presence of areas showing the accumulation of DAT immunolabeling (Figure 2N,R,V, arrowheads). Interestingly, these DAT-positive clumps, especially the larger ones that displayed neuropil-like morphology, were also α-synuclein immunopositive (Figure 2P,T,X, arrowheads). The co-localization between the DAT and α-synuclein was confirmed by performing an orthogonal z stack confocal acquisition of the immunostaining performed on the slice (Figure 3A–C). In particular, we observed that α-synuclein and DAT signals co-localized within positive clusters in the PD brain (Figure 3B,C, arrowheads), whereas the control subjects exhibited a diffuse pattern of co-localization (Figure 3A). Moreover, we observed that in the brain of patients affected by PD, the DAT co-localized with phospho-α-synuclein (Figure 3E–G), which is considered a key neuropathological hallmark of LB in synucleinopathies [37,38,39]. The phosphorylated form of α-synuclein was not detected in the brains of the control subjects (Figure 3D). For these experiments, negative controls were performed by incubating the tissue with the secondary anti-rabbit and anti-mouse antibodies for DAT and α-synuclein, respectively (Figure 2A–D).

### 2.3. Dopamine Transporter/α-Synuclein In Situ PLA in the Caudate Putamen of PD and Control Subjects

**Figure 4:**
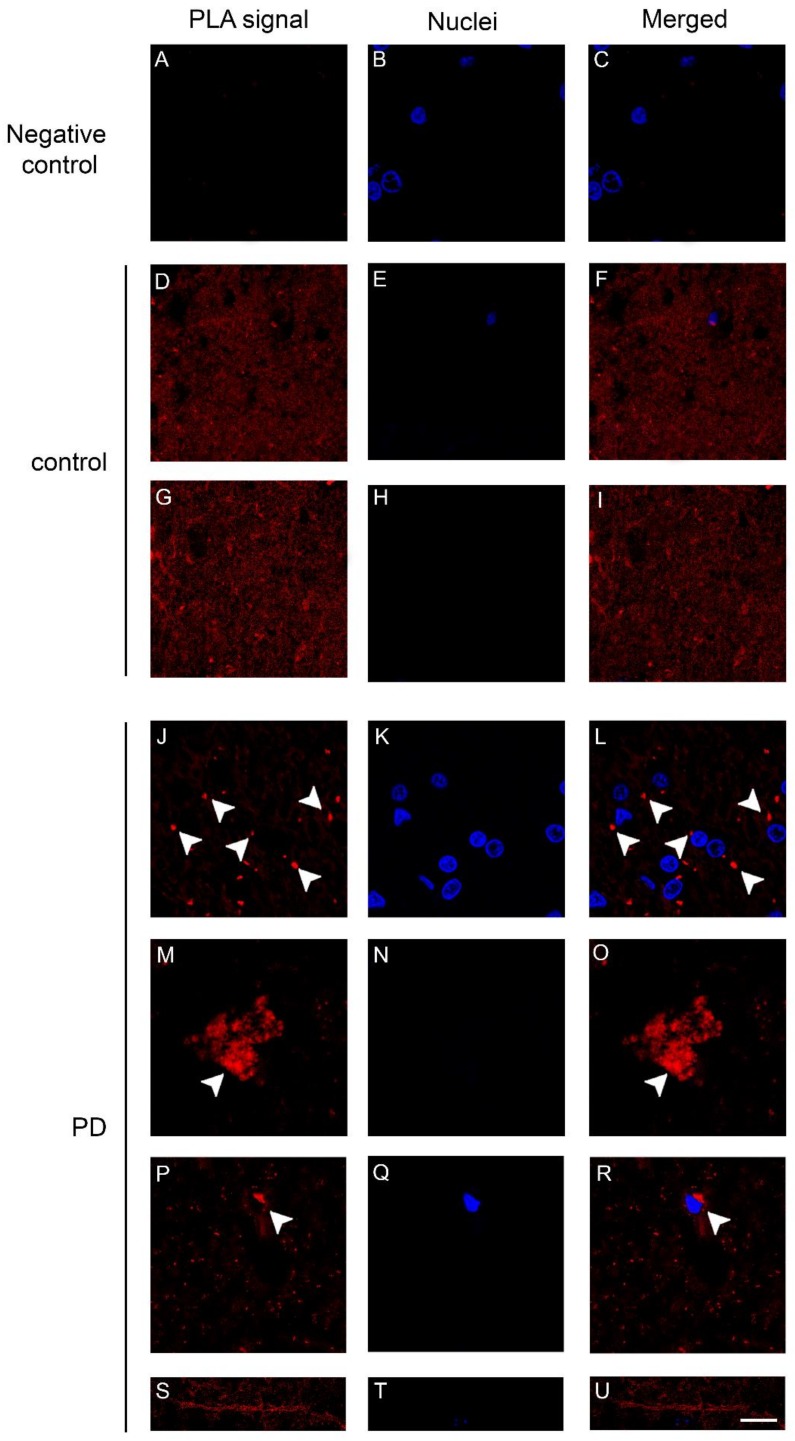
Dopamine transporter (DAT)/α-synuclein in situ PLA performed on the caudate putamen of PD patients and control subjects. (**A**–**C**) Panels showing the negative control, performed by omitting the DAT antibody, of the in situ PLA assayed on the caudate putamen of a control subject. The absence of the PLA-positive signal is indicative of the specificity of the assay; (**D**–**U**) Representative images showing DAT/α-synuclein in situ PLA performed on the caudate putamen of PD patients (**J**–**U**) and age-matched-controls (control, **D**–**I**). The presence of a PLA positivity as a red fluorescent signal is indicative of the interaction between the two proteins. Please note the marked redistribution of the PLA-positivity in the caudate putamen of PD samples (**L**,**O**,**R**) when compared to the control subjects (**F**,**I**). In particular, in the PD brains, the PLA signal was particularly abundant within big (**O**, arrowheads) and small (**L**,**R**, arrowheads) clumps or neuritic-like structures (**U**). Scale bar: 20 μm.

We previously described the occurrence of changes in the distribution of DAT/α-synuclein complexes in the substantia nigra and in the striatum of 12 month-old transgenic mice for C-terminally truncated α-synuclein [7] that reproduce early dopaminergic synaptic alterations and dysfunction [29]. Here, we used fluorescence-based in situ PLA to analyze the caudate putamen of PD patients and control subjects. The in situ PLA signal observed in the human brain was almost comparable with that observed in the human C-terminally truncated α-synuclein transgenic mouse model of PD [7]. The caudate putamen of PD and control samples showed the presence of PLA positive red dots indicative of the presence of DAT/α-synuclein complexes (Figure 4), but their distribution was significantly different between the two groups. Indeed, in the control subjects, the PLA-positive signal exhibited a diffuse distribution (Figure 4D–I), whereas in the PD brains, we observed the presence of PLA-positive complexes of different sizes, from small (Figure 4L,R, arrowheads) to very large ones (Figure 4O) and neuropil-like appearances. Interestingly, in the caudate putamen of one of the PD patients, we found different PLA positive LN-like structures (Figure 4U). In line with the immunolabeling data, the PLA positive signal appeared diminished in the PD subjects when compared to the controls. This may be related to the reduction of DAT and α-synuclein in the caudate putamen [25,26,27].

## 3. Discussion

The results of this study support the occurrence of a redistribution of DAT/ α-synuclein complexes in the brain of PD patients. While in the caudate putamen of the control subjects, DAT/α-synuclein complexes were found to display a dot-like appearance, with widespread distribution, we found that in the brains of patients affected by PD, they showed an apparent overall reduction, but were also clustered within several areas where most of them exhibited an increased size. Remarkably, in some areas of the PD caudate putamen, the DAT/α-synuclein PLA signal displayed a neuropil-like and filiform-shape neuritic-like morphology, supporting a participation of these complexes in disease neuropathology. By using double fluorescence immunohistochemistry, we observed that the DAT immunolabeling traced the distribution of in situ PLA within caudate putamen areas where α-synuclein immunolabeling was particularly intense within big dot-like inclusions. Moreover, these inclusions were found to be positive for phospho-α-synuclein, confirming their pathological nature [37,38,39]. Interestingly, these big DAT/α-synuclein complexes were reminiscent of those we described in the striatum of transgenic mice for C-terminally truncated α-synuclein [7]. The reduction of DAT/α-synuclein complexes observed in the brain of the PD subjects is in line with the results of our previous studies on this brain cohort [27] and suggests that a large number of DAT/α-synuclein complexes may have been lost along with dopamine (DA) neuron degeneration.

Several studies have demonstrated that α-synuclein interacts with and is involved in the regulation of DAT function in in vitro and in vivo models of PD [7,10,15,16,18,19,22,23]. The in situ PLA has been recently found to allow the detection of neuropathological features that differ from those classically observed by immunohistochemical methods in PD and LB dementia [27,30,31]. This evidence, together with the present findings, support that α-synuclein aggregation may lead to DA neuron derangement by disrupting the proper distribution of its synaptic and somatic interactants [7,10,30,36]. Therefore, the use of the in situ PLA can be pivotal in investigating the components of the neuropathological lesions of PD and to clarify its etiology. As an intrinsically disordered protein, α-synuclein has unique structural features that allow this protein to act as a hub in protein interaction networks [40,41]. Thus, the process of accumulation and aggregation of α-synuclein in the brain of PD patients could represent one of the causes of DAT redistribution and dysregulation [7]. Remarkably, although α-synuclein oligomers are not assembled through covalent bonds, they result in being highly stable and SDS-resistant [42]. Proteins, that like α-synuclein are disordered under physiological conditions or contain large unstructured regions, commonly interact with well-structured binding sites on other biomolecules [41,43]. These proteins can display unexpected interaction mechanisms mediated by large opposite net charges without requiring defined binding sites or interactions between specific individual residues that lead to the formation of high affinity complexes [43], a mechanism that could potentially explain the elevated stability of soluble α-synuclein oligomers. It could be feasible that this peculiar structural feature of α-synuclein may render the protein able to interact with other protein partners along the formation of high molecular weight aggregates. As a consequence, the subcellular localization of an α-synuclein interactome would be severely altered by its aggregation process and not solely because of the loss of function of the protein. The presence of a redistribution and size change of DAT/α-synuclein complexes in the caudate putamen of PD, when coupled to the results of our studies on experimental models showing that α-synuclein aggregation impairs DAT function by inducing its translocation from the plasma membrane to intracellular DAT/α-synuclein-positive inclusions [7,10], supports that the resulting impairment of DA turnover at dopaminergic synapses could play a pathological role in the early phases of α-synuclein deposition at striatal terminals in PD. For instance, 12-month old transgenic mice for the C-terminally truncated α-synuclein represent an ideal model for the study of the α-synuclein-related pathological changes in the very early pre-symptomatic phases of PD. Indeed, they exhibit a marked deposition of fibrillary α-synuclein aggregates in nigrostriatal dopaminergic neurons that is associated with striatal redistribution of synaptic proteins (DAT, Soluble N-ethylmaleimide-sensitive factor attachment protein receptor proteins (SNAREs) and synapsin III) and decreased basal and depolarization-dependent DA release in the absence of cell loss or motor impairment [29,44].

This hypothesis is in line with compelling evidence showing that nigrostriatal degeneration in PD proceeds with a retrograde pattern from the terminals of the caudate putamen to the soma of the substantia nigra and may be dependent on α-synuclein aggregation at synaptic sites [3,45]. In PD, the load of α-synuclein at synapses largely exceeds its amount within LB [46]. In addition, PET studies showed that at early symptomatic stages of the disease, a greater DAT loss is observed at the level of the axonal terminals when compared with cell bodies and axons of dopaminergic neurons [34]. This evidence is in agreement with findings showing that DAT imaging is indicative of early synaptic and axonal damage and does not reflect the number of viable neurons in the substantia nigra [47]. Neuropathological analysis of both the cell bodies and terminal areas of the nigrostriatal system suggests that at symptom onset, there is a moderate loss of at least 50% of fibers in the putamen that continue to express sufficient tyrosine hydroxylase (TH) and the DAT to be detected and a comparable loss of TH-positive stained cells in the substantia nigra [25]. This notwithstanding, although the loss of nigral TH-positive cells is more marked at the earliest time points, the loss of melanin containing neurons occurs more gradually over the course of the first decade following diagnosis, with differences in the number of TH-positive and melanin-positive neurons being preserved at all post-diagnostic time points. In parallel, the drop of TH and DAT immunolabeling progresses more severely [25]. Besides supporting that substantia nigra neurons may show different vulnerability thresholds along PD progression, these findings further confirm the occurrence of a retrograde degeneration pattern for dopaminergic neurons in this disorder. Our results showed that despite the massive loss of striatal DAT, in the late phases of PD, we could still detect neuropathological alterations such as big PLA-positive clumps containing this protein in association with α-synuclein in specific areas showing a marked accumulation of α-synuclein pathology. This evidence supports that the aberrant formation of DAT/α-synuclein complexes may contribute to DA neuron degeneration in PD. These findings pave the way for longitudinal in situ PLA studies on post mortem human brains and experimental models of PD that could be pivotal to determine the relevance of the redistribution of DAT/α-synuclein complexes in the pathogenesis of this disorder.

## 4. Material and Methods

### 4.1. Human Tissues

Paraffin embedded sections as well as fresh frozen tissue from six patients with idiopathic PD (age 79 ± 4) and five age-matched control subjects (age 80 ± 9) representing a subgroup of those studied in [27], kindly supplied by the Parkinson’s UK Brain Bank, a charity funded by Parkinson’s UK (Imperial College London, London, UK) were used for immunostainings and PLA, respectively. Samples from the PD patients showing the most severe and comparable α-synuclein/synapsin III neuropathology as assessed in our previous study [27] and the respective age matched controls were used. These patients showed different genders, age, disease duration, or age of onset, medications and post-mortem interval (PMI). For more detailed information about the subjects included in the study, please see Table 1. Sections from the caudate putamen and cerebellum of PD patients and age-matched control subjects were paraffin embedded and supplied at 5 µm thicknesses. The study on human brain samples was performed in accordance with the local clinical research regulations and obtained approvals from the Ethics Committee of Brescia District (NP no. 1537, 3 December 2013).

### 4.2. Immunohistochemistry

For immunohistochemical studies, we analyzed the caudate putamen and cerebellum sections from PD patients and age-matched control. Briefly, following deparaffinization and antigen retrieval with 10 mM sodium citrate buffer, sections were incubated for 1 h at room temperature (rt) in blocking solution made up by 2% *w/v* bovine serum albumin (BSA, Sigma Aldrich, St. Louis, MI, USA), 3% *v/v* normal goat serum (NGS, Sigma Aldrich), 0.3% Triton X-100 diluted in phosphate buffer saline (PBS) 0.1 M pH 7.4, and then with a rabbit polyclonal primary antibody recognizing the DAT (1:400, sc-14002, Santa Cruz Biotechnology, Dallas, TX, USA) or the mouse monoclonal primary antibody recognizing α-synuclein (1:500, MA5-12272, Syn211, Thermo Fisher Scientific, Waltham, MA, USA) diluted at an optimal working concentration in the above described blocking solution overnight (on) at 4 °C. Sections were then washed twice with 0.3% Triton X-100 diluted in PBS 0.1 M pH 7.4 and incubated with the opportune rabbit (1:1000, BA-1000, Vector Laboratories, Burlingame, CA, USA) or mouse (1:1000, AP124P, Thermo Fisher Scientific) biotin-conjugated secondary antibody diluted at optimal working concentrations for 45 min at rt. The immunostaining was visualized with an avidin-biotin system (Vector Laboratories, Burlingame, CA, USA) and 3,3-diaminobenzidine as the chromogen (DAB kit, Vector Laboratories). Counterstaining was performed using hematoxylin for 3 min and Eosin Y (0.5%) for 30 s. Sections were then washed and dehydrated before mounting using the Vectamount mounting medium (Vector Laboratories). For double labeling immunofluorescence staining, after the on incubation with the rabbit polyclonal primary antibody recognizing DAT (1:400, sc-14002, Santa Cruz Biotechnology), sections were washed with 0.3% Triton X-100 diluted in PBS 0.1 M pH 7.4 and incubated with the appropriate fluorophore-Alexa Fluor 488-conjugated rabbit secondary antibody (1:1000, 111-545-144, Jackson Immuno Research, West Grove, PA, USA) for 45 min at rt. This was followed by three washes in 0.3% Triton X-100 diluted in PBS 0.1 M at pH 7.4 and then by incubation with a second monoclonal mouse primary antibody recognizing α-synuclein (1:500, MA5-12272, Syn211, Thermo Fisher Scientific) or with a second monoclonal mouse primary antibody recognizing phospho-α-synuclein (1:400, ab184674, AbCam, Cambridge, UK) diluted at optimal working concentrations in blocking solution for 2 h at rt. Sections were finally incubated with the optimal Cy3-conjugated mouse secondary antibody (1:1000, 115-166-062, Jackson Immuno Research, West Grove, PA, USA) and then washed three times with 0.3% Triton X-100 diluted in PBS 0.1 M, pH 7.4 for 1 h at rt. Cell nuclei were then counterstained with Hoechst 33258 (Sigma Aldrich), and finally, sections were mounted using the Vectashield mounting medium (Vector Laboratories).

### 4.3. In Situ PLA

The in situ PLA allows the detection of protein-protein interactions in situ in intact tissues [7,27,30,31,32,33,48]. For the fluorescence human in situ PLA studies, we analyzed paraffin embedded brain sections from the caudate putamen of PD patients and age-matched controls by using the Duolink assay kit (O-Link Bioscience, Uppsala, Sweden) with a protocol adapted from the manufacturer’s instructions. Briefly, following deparaffinization and antigen retrieval with 10 mM sodium citrate buffer, sections were incubated in a blocking solution (provided by the kit) for 1 h at rt and then with the primary antibodies recognizing DAT (sc-14002, Santa Cruz Biotechnology), and α-synuclein (MA5-12272, Syn211, Thermo Fisher Scientific) at 1:100 dilution at 4 °C. On the following day, samples were washed and then incubated with a PLA probe solution for 1 h at rt. Sections were then washed and incubated with the ligation solution for 45 min at 37 °C, and then with the amplification solution at 37 °C for 100 min. Finally, cell nuclei were then counterstained with Hoechst 33258 (Sigma-Aldrich), and sections were mounted using the Vectashield mounting medium (Vector Laboratories).

### 4.4. Bright Field and Confocal Microscopy

Sections were observed by means of either an inverted light/epifluorescence microscope (Olympus BX41; Olympus, Tokyo, Japan) or a Zeiss confocal laser microscope LSM 880 (Carl Zeiss, Oberkochen, Germany) with the laser set on λ = 405–488–543 nm for the double immunostaining and on λ = 405–594 nm for the in situ PLA and the height of the sections scanning = ~1 μm. Images (512 × 512 pixels) were then reconstructed using Zen lite 2.3 (Carl Zeiss) and Adobe Photoshop CC (Adobe system, San Jose, CA, USA) software.

## Figures and Tables

**Table 1:** List of patients affected by PD and the control subjects used for the experiments. Each X indicates the samples used for the different immune-based techniques.

	Case	Age	Sex	Onset	Duration	Drugs	PMI	Experiments		
								IHC	IF	PLA
PD	PD020	75	M	42	34	Sinemet, Artane, Selegiline, Pergolide Domperidone, Apomorphine, Quetiapine Bromocriptine	2	X	X	X
	PD045	80	M	60	19	Sinemet, Ropinirole Selegiline, Entacapone Tolcapone, Cabergoline	16	X	X	X
	PD050	82	F	68	14	Sinemet, Selegiline Entacapone, Pergolide	18	X	X	X
	PD081	73	M	65	9	Madopar, Amantadine Amitriptyline, Haloperidol	19		X	X
	PD093	81	F	67	14	Madopar, Cabergoline, Sulpiride, Selegiline, Olanzapine, Amantadine	22	X	X	X
	PD099	82	M	72	11	Pramipexole, Benzhexol, Sinemet, Madopar	10	X		X
Controls	PDC022	65	M				12	X	X	X
	PDC028	84	F				11	X	X	X
	PDC029	82	M				48		X	X
	PDC034	90	M				12		X	X
	C026	78	F				33	X	X	X
